# Analysis of Age-Dependent Alterations in Excitability Properties of CA1 Pyramidal Neurons in an APPPS1 Model of Alzheimer’s Disease

**DOI:** 10.3389/fnagi.2021.668948

**Published:** 2021-06-11

**Authors:** Paola Vitale, Ana Rita Salgueiro-Pereira, Carmen Alina Lupascu, Michael Willem, Rosanna Migliore, Michele Migliore, Hélène Marie

**Affiliations:** ^1^Institute of Biophysics, National Research Council, Palermo, Italy; ^2^Université Côte d’Azur, CNRS, IPMC, Valbonne, France; ^3^Biomedical Center, Ludwig Maximilian University of Munich, Munich, Germany

**Keywords:** hippocampus, electrophysiological features, clustering analysis, computational modeling, amyloidopathy

## Abstract

Age-dependent accumulation of amyloid-β, provoking increasing brain amyloidopathy, triggers abnormal patterns of neuron activity and circuit synchronization in Alzheimer’s disease (AD) as observed in human AD patients and AD mouse models. Recent studies on AD mouse models, mimicking this age-dependent amyloidopathy, identified alterations in CA1 neuron excitability. However, these models generally also overexpress mutated amyloid precursor protein (APP) and presenilin 1 (PS1) and there is a lack of a clear correlation of neuronal excitability alterations with progressive amyloidopathy. The active development of computational models of AD points out the need of collecting such experimental data to build a reliable disease model exhibiting AD-like disease progression. We therefore used the feature extraction tool of the Human Brain Project (HBP) Brain Simulation Platform to systematically analyze the excitability profile of CA1 pyramidal neuron in the APPPS1 mouse model. We identified specific features of neuron excitability that best correlate either with over-expression of mutated APP and PS1 or increasing Aβ amyloidopathy. Notably, we report strong alterations in membrane time constant and action potential width and weak alterations in firing behavior. Also, using a CA1 pyramidal neuron model, we evidence amyloidopathy-dependent alterations in *I*_*h*_. Finally, cluster analysis of these recordings showed that we could reliably assign a trace to its correct group, opening the door to a more refined, less variable analysis of AD-affected neurons. This inter-disciplinary analysis, bringing together experimentalists and modelers, helps to further unravel the neuronal mechanisms most affected by AD and to build a biologically plausible computational model of the AD brain.

## Introduction

Increasing accumulation of the beta-amyloid peptides (Aβ) in the brain, a pathological phenomenon termed progressive amyloidopathy, is thought to be an important factor underlying the decline of cognitive functions in Alzheimer’s disease (AD) (Selkoe and Hardy, 2016). In human AD patients, this Aβ accumulation is particularly precocious in the hippocampus, a key brain region involved in memory processing, and worsens with aging. For this reason, Aβ-dependent hippocampus dysfunction has been intensively investigated in pre-clinical studies to identify the basis of memory loss in AD. Progressive age-dependent accumulation of Aβ with the formation of amyloid plaques, as observed in AD patients, is well reproduced in a number of transgenic mouse lines (Morrissette et al., 2009; Ashe and Zahs, 2010). To generate these models, the amyloid precursor protein (APP) is generally overexpressed as a transgene alone or together with another transgene expressing presenilin 1 (PS1), a member of the γ-secretase complex responsible for the cleavage of APP and the production of Aβ. To increase the generation of progressive amyloidopathy in these models, both APP and PS1 harbor mutations that are found in familial Alzheimer’s disease. These pre-clinical models exhibit age-dependent hippocampus dysfunction that correlate with hippocampus-dependent memory loss (Gong et al., 2004; Trinchese et al., 2004; Ma et al., 2012; Kartalou et al., 2020). These models are thus pertinent to investigate the impact of AD-like progressive amyloidopathy on hippocampus function.

In these models, most neurophysiological studies focused on the analysis of synapse function in the hippocampus. These studies identified alterations in several forms of excitatory synapse plasticity, such as long-term potentiation and depression (reviewed in Marchetti and Marie, 2011). More recently, other aspects of hippocampus activity are being investigated in the context of AD, in particular disturbances in intrinsic excitability and network synchrony at the basis of brain oscillatory activities, two other facets of hippocampus function crucial for adequate memory processing (Hanslmayr et al., 2019). Changes in network activity have been observed in human AD patients, who also suffer from a higher incidence of seizures (Amatniek et al., 2006; Scarmeas et al., 2009). Correlating with these clinical observations, work in AD mouse models evidences alterations in hippocampus network synchrony (Goutagny et al., 2013; Cayzac et al., 2015). At a single neuron level, neuron excitability has been characterized by measuring either passive membrane properties, single action potential (AP) kinetics or intrinsic excitability properties of hippocampal CA1 pyramidal neurons in a few of these APP or APP/PS1 over-expressing models when they were at a particular stage of progressive amyloidopathy (Brown et al., 2011; Kaczorowski et al., 2011; Verret et al., 2012; Wykes et al., 2012; Kerrigan et al., 2014; Šišková et al., 2014; Tamagnini et al., 2015). Only three of these studies analyzed specific aspects of neuronal excitability profiles at two ages in the same model to correlate progressive Aβ accumulation to alterations in some of these parameters (Brown et al., 2011; Kaczorowski et al., 2011; Šišková et al., 2014). When these studies are compared, no clear consensus emerges regarding alterations in the different parameters of neuron excitability. This could be at least partially due to the fact that each study investigated a different mouse model of amyloidopathy, moreover at different ages, representing different levels of amyloidopathy progression.

A thorough age-dependent investigation of these neuronal excitability parameters in the same model for correlative analysis of phenotypes with progressing Aβ amyloidopathy has yet to be reported. This type of analysis is particularly relevant for several reasons. First, when reporting phenotypes of these mice, authors usually point to Aβ accumulation as the cause of these phenotypes. Yet, there is increasing evidence that both APP and PS1 proteins *per se*, regulate neuron function even under physiological conditions (Saura et al., 2004; Weyer et al., 2011; Pousinha et al., 2017; Barthet et al., 2018; Lee et al., 2020). Overexpression of these proteins in the mouse models could therefore in itself perturb the different parameters analyzed, but these phenotypes should be observed independently of Aβ accumulation. It is thus of interest to more clearly correlate alterations in neuronal excitability to progression of Aβ amyloidopathy that occurs with aging in these models. This type of correlative analysis cannot definitely assign functional alterations to Aβ accumulation, but it should help us pinpoint phenotypes that are most likely to be due to this neuropathological hallmark. With the active development of computational modeling of AD (Golriz Khatami et al., 2019), it will be necessary to obtain such experimental data to build a reliable disease model exhibiting AD-like disease progression.

To this end, we performed a patch-clamp analysis of the passive membrane properties, single action potential kinetics (AP) and intrinsic excitability profile of CA1 pyramidal neurons of the hippocampus in an APPPS1 mouse model at three different ages representing amyloidopathy free, weak amyloidopathy and strong amyloidopathy stages of the disease and compared them to control wild-type (WT) littermates. This APPPS1 mouse model (Radde et al., 2006) is particularly useful to study the progression of amyloidopathy as these mice exhibit alterations in Aβ CSF levels, Aβ brain load and Tau CSF levels in a temporal sequence and magnitude of Aβ and Tau changes observed in the CSF of patients with sporadic and dominantly inherited AD (Maia et al., 2013). We used the feature extraction tool available on the Brain Simulation Platform of the Human Brain Project to analyze these data and report the results obtained for 14 features linked to either passive membrane properties, single action potential kinetics (AP) or intrinsic excitability profile. We also took advantage of the latest CA1 pyramidal neuron model developed by Migliore et al. (2018) to predict additional alterations in underlying currents that were not directly accessible by feature extraction of the recorded traces. Finally, using these extracted features, we performed a cluster analysis to predict if we could assign traces of the different conditions to their respective groups.

## Materials and Methods

### Animals

APPPS1 mice carrying transgenes expressing human APP bearing the KM670/671NL “Swedish” mutation and human PS1 bearing the L166P mutation under control of the Thy1 promotor were used in this study (Radde et al., 2006)^1^. The animals were generated and maintained on a C57BL/6J genetic background (Charles River, France). APP/PS1 and WT male littermates were used at three ages: 1 month (3–4 weeks), 3–4 months, and 9–10 months of age. All experiments were done according to policies on the care and use of laboratory animals of the European Committees Council Directive (86/609/EEC). The protocols were approved by the French Research Ministry following evaluation by a specialized ethics committee (APAFIS#6855-20 16091615385487 v5). All efforts were made to minimize animal suffering and reduce the number of animals used. The animals were housed six per cage under controlled laboratory conditions with a 12-h dark light cycle, a temperature of 22 ± 2°C. Animals had free access to standard rodent diet and tap water.

### Biochemical Analysis of Aβ Load

Four APPPS1 mice of 1 month of age, 6 APPPS1 mice of 3–4 months of age, 5 APPPS1 mice of 9–10 months of age and 4 WT mice of 9–10 months of age were sacrificed and their hippocampi were dissected out and snap-frozen in liquid nitrogen to process for biochemical analysis of Aβ load. Briefly, DEA (0.2% Diethylamine in NaCl 50 mM, pH 10) and RIPA lysates [Tris-HCl (pH 7.5) 20 mM, NaCl 150 mM, Na_2_EDTA 1 mM, NP-40 1%, sodium deoxycholate 1%, sodium pyrophosphate 2.5 mM] were prepared from the frozen tissues. The later was centrifuged at 14,000 *g* (60 min at 4°C) and the remaining pellet was homogenized in 70% formic acid (FA fraction). The FA fraction was neutralized with 20 × 1 M Tris-HCl buffer at pH 9.5 and used for Aβ analysis. For Aβ detection by immunoblotting, proteins were separated on Tris-Tricine (10–20%, Thermo Fisher Scientific, Germany) gels, transferred to nitrocellulose membranes (0.1 μm, GE Healthcare, United States) which were boiled for 5 min in PBS and subsequently incubated with the blocking solution containing 0.2% I-Block (Thermo Fisher Scientific, Germany) and 0.1% Tween 20 (Merck, United States) in PBS for 1 h, followed by overnight incubation with 2 mg/ml 2D8 antibody in the blocking solution. The rat monoclonal 2D8 antibody against Aβ was described before (Shirotani et al., 2007). Antibody detection was performed using the corresponding anti-rat-IgG-HRP conjugated secondary antibody (Santa Cruz) and chemiluminescence detection reagent ECL (Thermo Fisher Scientific, Germany). Aβ contained in FA fractions was quantified by a sandwich immunoassay using the Meso Scale Aβ Triplex 6E10 plates and Discovery SECTOR Imager 2400 as described previously (Page et al., 2008). Samples were measured in triplicates.

### Electrophysiology

Mice were anesthetized using Ketamine 150 mg/kg/Xylazine 10 mg/kg solution before transcardiac perfusion with the oxygenated (95% O_2_/5% CO_2_) ice-cold sucrose cutting solution containing (in mM): KCl 2.5, NaH_2_PO_4_ 1.25, MgSO_4_ 10, CaCl_2_ 0.5, glucose 11, sucrose 234, NaHCO_3_ 26. The brain was immediately removed and the dissected hippocampus was mounted in an agar support and placed in the previous oxygenated ice-cold sucrose cutting solution. Horizontal slices of the dorsal hippocampus with 250 μm were obtained using standard procedure (Pousinha et al., 2019), placed in an aCSF chamber containing (in mM): NaCl 119, KCl 2.5, NaH_2_PO_4_ 1.25, MgSO_4_ 1.3, CaCl_2_ 2.5, NaHCO_3_ 26, glucose 11 at 37°C for 1 h and then kept at room temperature. All chemicals were from Sigma-Aldrich (Lyon, France).

For recordings, slices were transferred to a recording chamber containing continuous circulating (2 mL/min), oxygenated and warm (32°C) aCSF. Slices were visualized on an upright microscope with IR-DIC illumination and epi-fluorescence (Scientifica Ltd, Uckfield, United Kingdom). Current-clamp experiments were obtained using a Multiclamp 700B (Molecular Devices, Sunnyvale, CA, United States). Signals were collected and stored using a Digidata 1440 A converter and pCLAMP 10.2 software (Molecular Devices, Sunnyvale, CA, United States). Whole cell current clamp recordings were made using 4–6 MΩ fire-polished glass electrodes and a tight seal (>1 GΩ) on the cell body of the selected neuron was obtained. Internal solution contained (in mM): K-D-gluconate 135, NaCl 5, MgCl_2_ 2, HEPES 10, EGTA 0.5, MgATP 2, NaGTP 0.4. The resting membrane potential (Vm) was first measured in the absence of any spontaneous firing, and only cells more negative than –50 mV were considered. The number of abnormally depolarized neurons were not different in the various conditions tested (about 5% in each condition) and most likely reflected a technical issue such as poor seal. The firing frequency was obtained by clamping neurons at Vh = –65 mV and then injecting pulses of increased intensity in steps of 50 pA (from –200 to 400 pA, 400 ms duration).

### Feature Extraction

Electrophysiological features were extracted from individual experimental traces using the Feature extraction tool available on the Brain Simulation Platform of the Human Brain Project^2^, which exploits the open source Electrophysiological Feature Extraction Library (eFEL^3^). Based on our judgment and to allow comparison with previous works, we decided to select 14 features. A total of 1200 traces were analyzed, using 14 different current injections. They were organized in 92 sets of recordings, with 14 neurons from WT-1m animals, 12 neurons from WT-4m, 19 neurons from WT-10m, 11 neurons from AD-1m, 15 neurons from AD-4m, and 21 neurons from AD-10m. Recordings were obtained from 10 control mice (3 WT-1m, 3 WT-4m, and 4 WT-10m) and 9 APPPS1 mice (3 AD-1m, 3 AD-4m, and 3 AD-10m). To calculate spike times, action potential threshold was set at –10 mV.

### Computational Modeling

All simulations were carried out using the NEURON simulation environment (v7.7.2, Hines and Carnevale, 1997). For all simulations we used a reconstructed morphology of a mouse CA1 pyramidal neuron (from neuromorpho.org, cell id NMO_60522, Peng et al., 2016), including only passive properties and an *I*_*h*_ taken from a previous model of rat CA1 neurons (Migliore et al., 2018). Temperature was set at 34°C. On apical dendrites, the *I*_*h*_ channel was distributed following a sigmoidal increase as a function of distance from soma (Migliore et al., 2018). Model and simulation files will be uploaded to the ModelDB database^4^ (accession no. 266848).

### Classification Analysis

The Matlab Classification Learner app was used to perform the classification of the traces. A 10-fold cross validation was performed, training the models on 90% of the data and validating on the remaining part. Splitting during the validation process was done randomly, and after 10 repeats all samples have been left out once. The classifiers that gave the best accuracy in most of the cases were *Ensembles of Bagged* or *Boosted Trees*, which combines several weak learners to produce better predictive performance than using a single weak learner. *Bagging* (Bootstrap Aggregation) is used in order to reduce the variance of a decision tree. Several subsets of data from training sample are created by choosing randomly with replacement. Each collection of subsets was used to train their decision trees. *Boosting* is another ensemble technique to create a collection of predictors. With this method, learners are formed sequentially with early learners fitting simple models to the data and then analyzing the results for errors. When an input was misclassified, its weight was increased so that a next iteration can more likely classify it correctly. With both methods, a strong learner is formed at the end of the classification process.

### Statistical Analysis

Results in all figures are presented as box plots containing median, quartiles, minimum/maximum values and outliers, if present. Statistical tests were performed using built-in functions in Sigmaplot v13.0 or Graphprism packages. In the great majority of cases, data were normally distributed, and a student *t*-test was used to compare results for Control and AD groups independently for each age and current injection. For samples that did not follow a normal distribution, a Mann–Whitney Rank-sum test was used. No multiple pairwise comparisons were carried out for statistical analyses. Each group of age was tested independently from the others. For ELISA data analysis, the data followed a normal distribution and were analyzed with a two-way ANOVA followed by Tukey’s multiple comparisons. A difference with a *p* ≤ 0.05 was considered statistically significant. All statistics are presented in Supplementary Tables 1–4.

## Results

**FIGURE 1:**
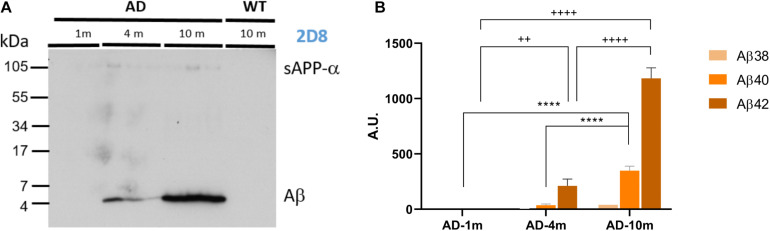
Analysis of Aβ load. **(A)** Immunoblot analysis of Aβ levels (FA fraction) in brains of APPPS1 mice (AD-1m, AD-4m, AD-10m) and WT control mice (WT-10m) (three mice of each group). While the increase in hippocampal Aβ is evident from the FA fractions obtained from AD-10m mice compared to AD-4m mice, no signal can be detected in AD-1m mice and WT-10m mice. **(B)** ELISA quantification of Aβ38/Aβ40/Aβ42 in FA extracts of AD-1m (four mice), AD-4m (six mice), and AD-10m (five mice). Statistical analysis was performed by Two-way ANOVA. Aβ40: ^*⁣*⁣**^p ≤ 0.0001; Aβ42: ^+ +^*p* ≤ 0.01; ^+ ⁣ + ⁣ + +^*p* ≤ 0.0001. See full statistical analysis in Supplementary Table 1.

To identify alterations in neuron excitability that correlate to different stages of Aβ amyloidopathy in the APPPS1 model, we recorded CA1 pyramidal neurons of mice at 1, 3–4, and 9–10 months of age. At these ages, the mice exhibit vastly different Aβ amyloidopathy profiles (Table 1; Radde et al., 2006; Serneels et al., 2009; Gengler et al., 2010; Maia et al., 2013; Sebastian Monasor et al., 2020). At 1 month of age, expression of human APP and PS1 transgenes in neurons is already high (starting at 2 weeks of age), but Aβ40, Aβ42 levels and the Aβ40/Aβ42 ratio are still very low. Amyloid plaques are completely absent and cognitive functions are still normal. This age group (APPPS1 and WT littermates) can be considered Aβ amyloidopathy free and is labeled WT-1m and AD-1m here after. At 3–4 months of age, Aβ accumulation is rising in the brain (both Aβ40 and Aβ42) and the ratio Aβ40/Aβ42 is fivefold that of control mice (Table 1). Amyloid plaques start to appear in the hippocampus, but these mice do not yet display hippocampus-dependent cognitive deficits. This age group (APPPS1 and WT littermates) thus exhibit markers mimicking weak Aβ amyloidopathy and is labeled WT-4m and AD-4m here after. At 9–10 months of age, strong Aβ amyloidopathy is reached in this model (Table 1) with very high brain levels of Aβ40 and Aβ42 and a stagnating Aβ40/Aβ42 ratio value. Strong amyloid plaque load is observed in the hippocampus and these mice are now exhibiting impaired cognitive functions when tested in hippocampus-dependent memory tasks (Radde et al., 2006; Serneels et al., 2009; Kartalou et al., 2020). This age group (APPPS1 and WT littermates) thus exhibit markers mimicking strong Aβ amyloidopathy, reaching levels observed in AD human patients, correlating to cognitive deficits, and is labeled WT-10m and AD-10m here after. To confirm that the chosen age groups displayed increasing Aβ amyloidopathy in the hippocampus, we measured Aβ load by extracting the FA fraction (RIPA insoluble) of these hippocampi. We used this fraction to detect Aβ by immunoblotting with the 2D8 antibody (Figure 1A) and quantified the levels of three main forms of Aβ (Aβ38, Aβ40, and Aβ42) by ELISA (Figure 1B). As expected, the AD-1m did not exhibit any accumulation of Aβ. In AD-4m mice, Aβ load started to rise, especially the Aβ42 form. In AD-10m, there was strong accumulation of Aβ, both of the Aβ40 and Aβ42 forms, which is in accordance with the original observation in this transgenic mouse line (Radde et al., 2006). No Aβ was detected in WT-10m mice by immunoblotting (Figure 1A) nor by ELISA (data not shown).

**TABLE 1:** Stages of amyloidopathy in APPPS1 mice.

AD marker	Aβ40 levels in brain	Aβ42 levels in brain	Aβ40/Aβ42 ratio	Amyloid plaques load in hippocampus	Cognitive impairment
Age					
*1 month*	Very low	Very low	∼1.5	None	No
*3–4 months*	High (∼600 times 1 month value)	High (∼2500 times 1 month value)	∼5	Modest	No
*9–10 months*	Very high (∼3000 times 1 month value)	Very high (∼15000 times 1 month value)	∼4.5	Strong	Yes

*Stages of amyloidopathy in APPPS1 mice at the 1, 3–4, and 9–10 months of age, representing no amyloidopathy, weak amyloidopathy and strong amyloidopathy stages, respectively. Data were extracted from previous analysis of this APPPS1 model (Radde et al., 2006; Serneels et al., 2009; Gengler et al., 2010; Maia et al., 2013; Sebastian Monasor et al., 2020). Note that these data are for whole brain values. We provide additional values of Aβ38, Aβ40, and Aβ42 levels obtained in hippocampi of these mice at these different ages in Figure 1.*

For these three age groups, we recorded the excitability profile of hippocampal CA1 pyramidal neurons in response to increasing somatic current injections by whole-cell patch clamp electrophysiology. We performed an analysis of the electrophysiological traces (see section “Materials and Methods”), and in the following subsections we present the results for selected features relative to passive membrane properties, the first action potential in a train, and the firing behavior. Results for features not discussed in the main text are reported in Supplementary Tables 2–4.

### Membrane Time Constant Is Differentially Affected in Aβ Amyloidopathy-Free Mice and in Mice With Strong Aβ Amyloidopathy

**FIGURE 2:**
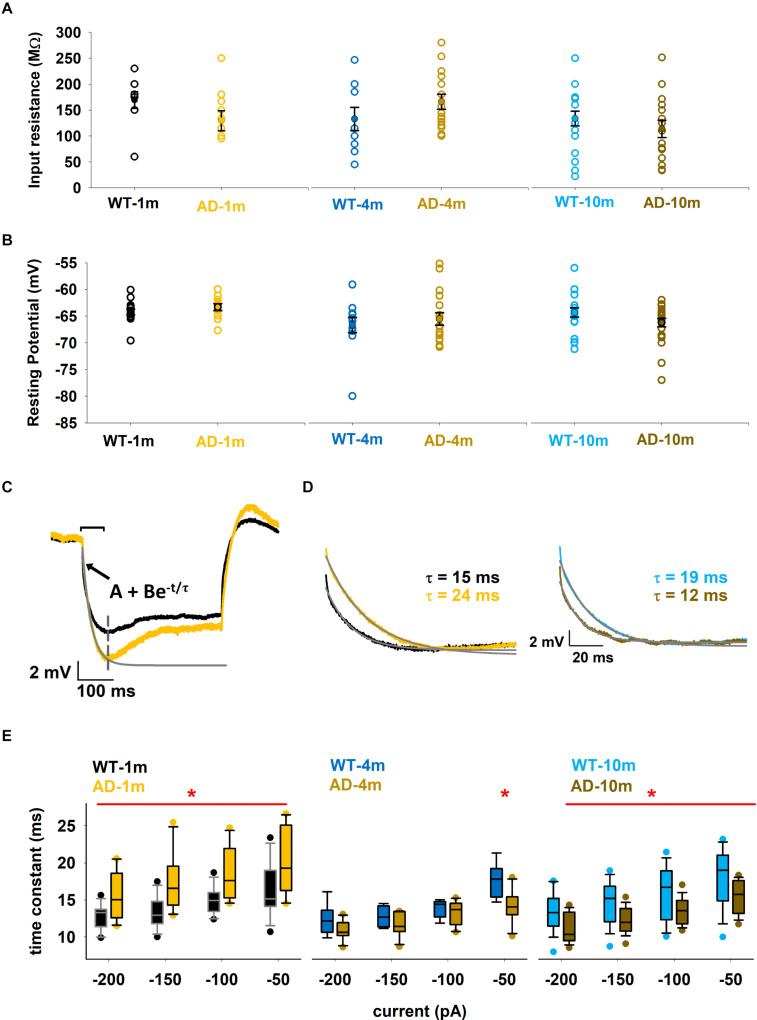
Differences in passive properties. **(A)** Input resistance of individual neurons for the different conditions. Average values are shown as closed symbols. **(B)** Native resting potential of individual neurons for the different conditions. Average values are shown as closed symbols. **(C)** Typical recordings for a –50 pA current injection from a WT-1m (black trace) or AD-1m (orange) mice. The gray line represents the exponential time course best fitting the initial part of the experimental trace and used to calculate τ. The inset shows the equation used for the fit. **(D)** Typical traces (*left*, WT-1m and AD-1m; *right* WT-10m and AD-10m) used for the calculation of the membrane time constant for a –50 pA input current. Gray lines represent the exponential decay used to calculate τ values; experimental traces have been rescaled to have the same minimum value. **(E)** Box plot of membrane time constant as a function of the current injection. The red line and the * symbol indicate for which current the values for WT and AD groups were significantly different (*p* ≤ 0.05). See full statistical analysis for all parameters in Supplementary Table 2.

We first analyzed the passive membrane properties of CA1 pyramidal neurons in each condition by analyzing the input resistance (*R*_*in*_), Resting membrane potential (RMP, measured in the experiments), and the membrane time constant. These measures allow us to identify if neurons have modified their general response to current changes, as would occur in a simple passive electrical circuit. Alterations in these passive properties can influence the integration of incoming electric signals. Values of these measures are reported in Figure 2 for all groups. We calculated *R*_*in*_ in two different ways, both based on Ohm’s law: (1) using the native resting potential of a neuron and the holding current needed to bring it to –65 mV; (2) using the steady-state hyperpolarization reached during small negative somatic current injections. In both cases, we did not observe significant differences between genotypes in each age group (*p*_*Rin*_ = 0.133, 0.207, and 0.369 and *p*_*RMP*_ = 0.291, 0.516, and 0.06 after *t*-test, for 1, 3–4, and 9–10 months respectively). In Figure 2A we plot the values obtained using the first method. RMP was also stable within the different age groups (Figure 2B). The membrane time constant was calculated for different negative current injections (–200, –150, –100, and –50 pA) by fitting the first part of the membrane potential, from the start of the current injection up to the peak of the sag, as schematically shown in Figure 2C for two typical traces from WT-1m (Figure 2C, black line) and AD-1m (Figure 2C, orange line) in response to a –50 pA current injection. By calculating the time constant at different current steps, we had the possibility to discover a feature that has been so far neglected in most experiments, namely a modulation of the membrane time constant with the hyperpolarization level. Typical traces obtained for a –50 pA injection are plotted in Figure 2D, and suggest that AD-1m mice exhibited a significantly slower membrane time constant compared to WT-1m littermates (Figure 2D, left). This alteration normalized in AD-4m mice, and became significantly faster in AD-10m mice, with respect to their aged-matched WT littermates (Figure 2D, right). Quantified results are reported in Figure 2E. Together, these data suggest that this membrane property is differently perturbed in young AD mice (without observable Aβ amyloidopathy) and in old mice (with strong Aβ amyloidopathy), with faster membrane time constant correlating with increased Aβ load.

### I_*h*_ Current Is Affected in APPPS1 Mice With Strong Aβ Amyloidopathy

**FIGURE 3:**
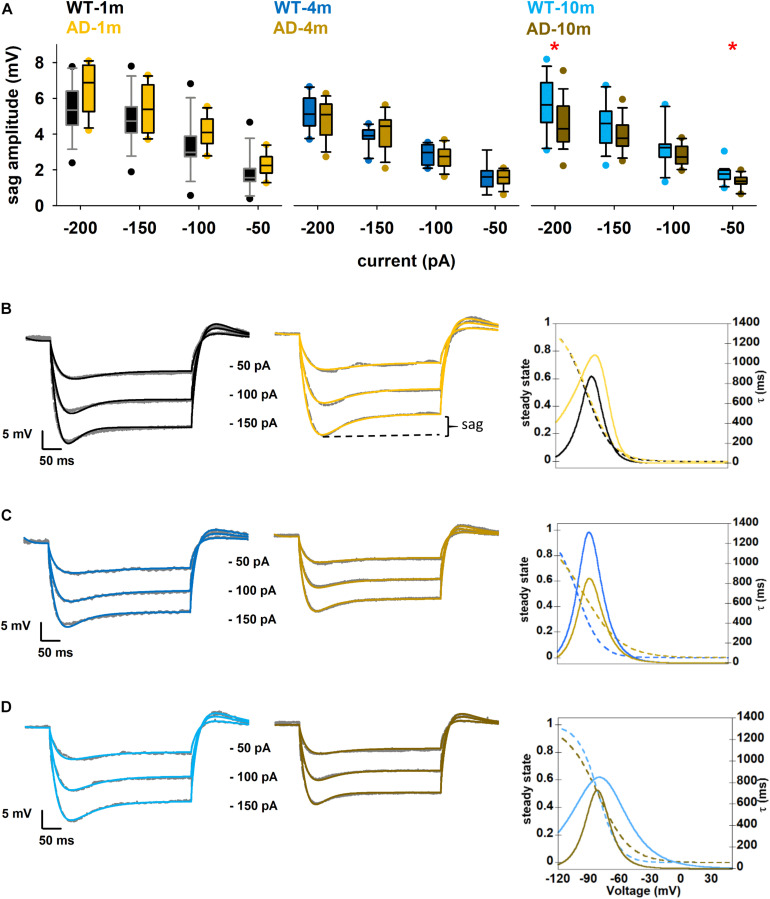
Differences in *I*_*h*_ current properties. **(A)** Box plot of sag amplitude as a function of current injection; See full statistical analysis for sag amplitude in Supplementary Table 2. **(B–D)** Comparison between model (colored) and experimental (gray) traces under Control and AD conditions for 1, 3–4, and 9–10 months, panels **(B–D)**, respectively; (*left and middle*) best fit of experimental traces for each condition; (*right*) kinetic curve for each genotype. Dotted lines represent steady state and solid lines represent time constant of activation of *I*_*h*_ current. *p ≤ 0.05.

In Figure 2E one can also observe that, for all genotypes and ages, the time constant becomes approximately 20% faster with stronger hyperpolarization. These data provide a clear indication of the involvement of a shunting, hyperpolarization activated, current in these neurons. Furthermore, under all conditions, we also observed a prominent sag in all hyperpolarizing current injections. A sag is defined as the difference between the minimal voltage reached during a hyperpolarizing current step and the steady state voltage at the end of the stimulus (Figure 3B), and it is a typical characteristic of cells possessing the *I*_*h*_ current. For this reason, we decided to investigate in more detail the sag amplitude, as it can provide an indirect measure of alterations in *I*_*h*_ peak value or kinetics. A box plot of sag values as a function of hyperpolarizing current injection for each age group is shown in Figure 3A. Typical examples of traces exhibiting a sag are plotted in Figures 3B–D. The AD-1m and AD-4m mice did not show any difference in the sag amplitude (Figure 3A, left and middle panels, *p* > *0.05* in all cases). AD-10m mice (Figure 3A, right panel) exhibited a significant difference for –200 pA (5.6 ± 0.3 in WT-10m vs. 4.5 ± 0.3 in AD-10m, *p* = 0.034) and at –50 pA (1.7 ± 0.1 in WT-10m vs. 1.4 ± 0.1 in AD-10m, *p* = 0.013). This difference could be caused by modifications in *I*_*h*_ current.

Obtaining direct measures of *I*_*h*_ kinetic properties would have been technically challenging in the context of the experiments carried out in this work. For this reason, in order to figure out which *I*_*h*_ kinetic property might be affected in AD-10m neurons, we decided instead to implement a computational model using a three-dimensional morphological reconstruction of a mouse CA1 pyramidal neuron, including only passive properties and *I*_*h*_. We started from an *I*_*h*_ model file developed for rats (Migliore et al., 2018, modelDB acc.n. 244688 and HBP Live Papers^5^) and previously validated against a number of experimental findings (Migliore and Migliore, 2012). The kinetic parameters were optimized for mice under control conditions. As reference experimental traces, we used typical recordings at –150, –100, and –50 pA. The standard *Run Fitter* tool available in NEURON was used to simultaneously optimize the membrane resistance (R_*m*_), the leak reversal potential (*e_pas*), the *I*_*h*_ peak conductance and the kinetic parameters modeling the activation curve (the shape factor, *kl* and *Vl_1__/__2_*,) and its time constant (*Vt_1__/__2_, a0t*, *zetat*, and *gmt*). The optimization was carried out to find the best fit of a typical set of experimental traces for each condition. As can be seen in the left and middle panels of Figure 3B (for 1 m animals), Figure 3C (4 m animals), and Figure 3D (10 m animals), the optimizations converged into a good solution, with an error lower than 0.3 mV. The final parameters are reported in Table 2, and the *I*_*h*_ kinetics for each case is plotted in the right panels of Figures 3B–D. The role of *I*_*h*_ in modulating subthreshold membrane potential is demonstrated in Supplementary Figure 1, where we show the time constant for models in which we simulated the application of an *I*_*h*_ blocker.

**TABLE 2:** Optimized *I*_*h*_ model parameters.

	Rm (Ω/cm^2^)	e_pas (mV)	Peak I_*h*_ conduct. (S/cm^2^)	Vl_1__/__2_ (mV)	kl (mV)	Vt_1__/__2_ (mV)	a0t (ms^–1^)	zetat	gmt
*WT-1m*	29400	–88.28	1.57e-5	–89.85	–12.48	–83.43	3.02e-3	5.42	0.437
*AD-1m*	29400	–86.18	1.55e-5	–88.73	–13.08	–72.64	3.00e-3	4.47	0.181
*WT-4m*	21100	–89.68	1.39e-5	–99.94	–11.28	–89.72	2.00e-3	4.93	0.557
*AD-4m*	20700	–92.29	2.47e-5	–93.86	–18.92	–89.85	3.11e-3	4.96	0.582
*WT-10m*	28200	–87.48	1.55e-5	–80.86	–10.00	–76.85	3.10e-3	2.22	0.439
*AD-10m*	26400	–88.61	2.35e-5	–81.21	−15.62	–82.22	3.59e-3	5.00	0.514

*Model parameters that were optimized to fit *I*_*h*_ current in such a way to reproduce experimental traces. *Rm*, membrane specific resistance; *e_pas*, leak reversal potential; *Peak I_*h*_ conduct*, peak conductance of the *I*_*h*_ channel; *Vl*_1/2_ and *Vt*_1/2_, the voltage at which the activation curves is 0.5; *kl*, slope of the activation curve; *a0t, zetat* and *gmt*, kinetic parameters related to the height, width, and shape of the time constant for activation, respectively. In red, parameters that were substantially different in WT-10m and AD-10m neurons and can be responsible for the difference in the sag amplitude.*

To try to explain the alterations in the sag amplitude by alterations in *I*_*h*_ kinetics, we evaluated differences between WT-10m and AD-10m cells, in the physiological voltage range above –90 mV (Figure 3D). The model (see Table 2) for the AD-10m traces, suggested an approximately 50% increase in the peak *I*_*h*_ channel conductance and an activation kinetics (*kl*) 50% smoother than in WT-10m. These differences will generate opposite effects in the overall *I*_*h*_ current, and explain why for intermediate current injections (–100 and –150 pA) there was no difference in the average sag amplitude. Furthermore, for small hyperpolarizing currents (i.e., approximately above –80 mV), the activation time constant was much faster under AD conditions. Taken together, the model suggests that in AD-10m mice the amyloidopathy correlates with an increase of the *I*_*h*_ peak conductance, and a faster channel activation. Of note, the model suggested no alterations in *I*_*h*_ parameters for AD-1m mice, correlating with normal sag amplitudes at this age. However, in AD-4m mice the model suggested an increase in the peak *I*_*h*_ channel conductance and a smoother *I*_*h*_ activation kinetics (Table 2), although with a sag amplitude similar to control (Figure 3A). This latter effect could be explained with transitory compensatory mechanisms that are lost with aging and amyloidopathy progression.

### Action Potential Width Is Differently Affected in the Different Age Groups of APPPS1 Mice

**FIGURE 4:**
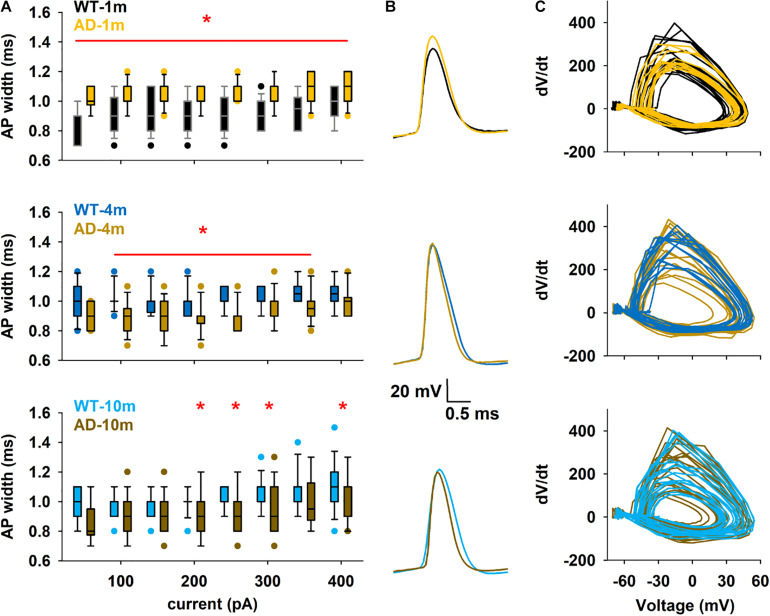
Differences in the first Action Potential of train. **(A)** Box plot of first AP width in the different conditions as a function of current injection. The red line and symbol in each plot indicate the currents for which the average values were significantly different. *p ≤ 0.05. **(B)** Comparison of typical first APs under different conditions for a 300 pA input current. **(C)** Phase plots for the first AP for a 300 pA current injection in the different conditions, calculated from all traces at 300 pA. See full statistical analysis for single AP analysis in Supplementary Table 3.

Modifications of single AP kinetics could strongly influence information transfer in the brain. We thus next focused our attention on the shape of the first AP generated by positive current injections (50–400 pA). Out of the different features analyzed for this AP (see Supplementary Table 3 for full analysis data), AP width of APPPS1 mice showed significant differences at all ages, and we noticed a reversal of this alteration correlating with progression of Aβ amyloidopathy (Figures 4A–C). Indeed, while AD-1m mice exhibited significantly wider AP width, this phenotype was opposite in AD-4m and AD-10m mice, where AP width was significantly narrower than the respective WT control littermates.

Additional analysis of AP shape was performed to investigate if modifications in Na^+^ channels function could be evidenced and responsible for this modification in AP width. For this purpose, we constructed phase-plane plots of dV/dt vs. V for the first AP in a train generated during 300 pA current steps, and analyzed the corresponding average dV/dt values (Figures 4B,C). No difference was observed in the different groups (*p* = 0.548, 0.710, 0.955 after *t*-test for 1, 3–4, and 9–10 months respectively), suggesting that sodium channel function is not altered at any of these ages in this mouse model (Supplementary Table 3). Our electrophysiological recordings did not allow us to analyze potential alterations in the function of potassium channels, which could also modify AP width.

### A Few Parameters of Firing Behavior Are Perturbed in APPPS1 Mice

We next analyzed firing behavior of these neurons, i.e., how they respond to increasing depolarizing current with trains of APs. Alterations in the normal firing behavior of neurons will modify information processing in the brain. We found that many electrophysiological features of this firing behavior, were not perturbed in the AD model over the entire range of current and ages tested (see Supplementary Table 4 for results on additional features). We do not discuss these features here further.

**FIGURE 5:**
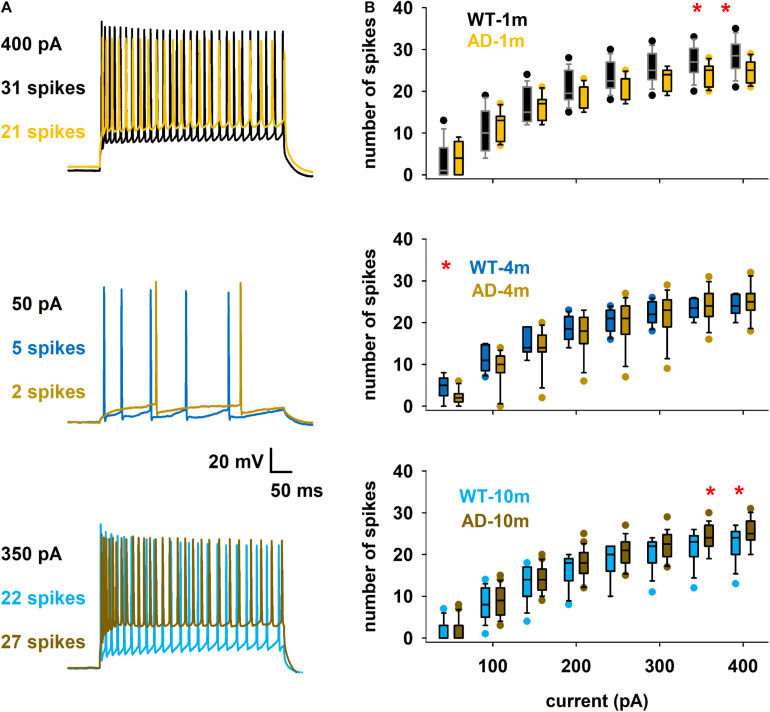
Differences in spike count. **(A)** Overlap of typical traces for the different conditions. **(B)** Box plot of number of spikes as a function of somatic current injection. The red symbols in the graphs indicate currents for which the average values were significantly different. *p ≤ 0.05. See full statistical analysis for these features in Supplementary Table 4.

**FIGURE 6:**
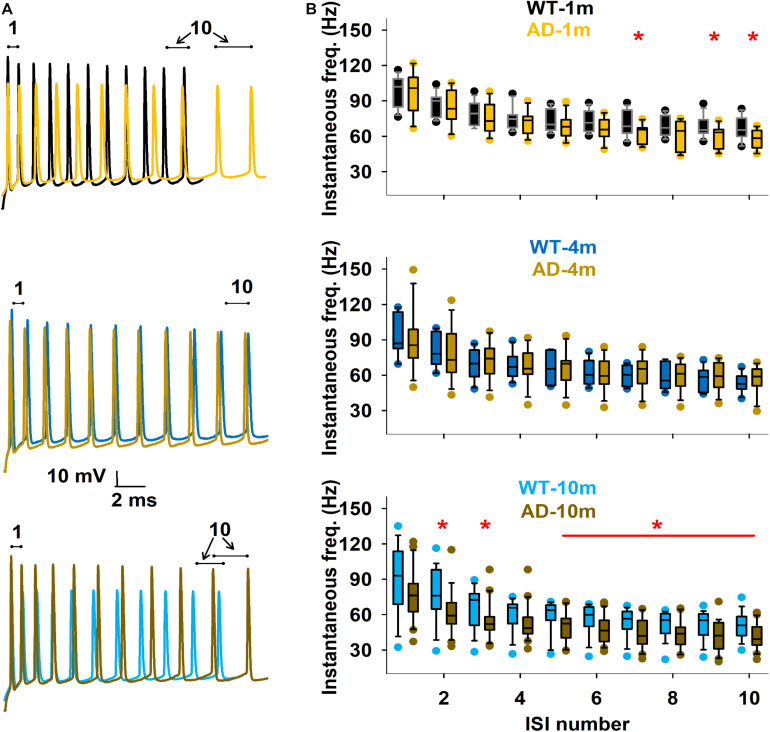
Differences in firing frequency. **(A)** Enlargement of the traces to illustrate first 11 spikes in typical experimental traces at 300 pA for each age, under Control and AD conditions. **(B)** Box plot of instantaneous firing frequency calculated from the first 10 inter-spike intervals, for the different conditions. The red line and symbols in the graphs indicate currents for which the average values were significantly different. *p ≤ 0.05. See full statistical analysis for these features in Supplementary Table 4.

We describe here features that were evaluated in other AD models in previous studies (Brown et al., 2011; Kerrigan et al., 2014; Tamagnini et al., 2015), that are the spike count (Figures 5A,B) and the instantaneous frequency (at 300 pA injection step, Figures 6A,B). We did not evidence any major alterations in spike count for most comparisons (Figure 5B). We however observed that spike count was lower at high current steps in AD-1m mice, suggesting lower excitability at this age. This phenotype normalized in AD-4m mice and reversed AD-10m with spike count becoming higher at high current steps in these mice, suggesting higher excitability. As these long high current steps do not reflect natural patterns of activation *in vivo*, we also analyzed instantaneous frequency at 300 pA for the first 11 spikes for each group. This instantaneous frequency analysis is a feature that is more relevant to *in vivo*-like situations. The instantaneous frequency was significantly lower than WT littermates in AD-1m mice from pulse 7 to 10, normalized in AD-4m mice and then was significantly lower again in AD-10m mice, but this time for most pulses analyzed. Together these data suggest that AD-1m and AD-10m neurons exhibit different firing behaviors from their WT littermates.

### Trace Classification

Finally, we hypothesized that the variability of the experimental traces under the different conditions may be caused by the differential impact of ongoing amyloidopathy on individual neurons. This could result in a significant number of neurons in APPPS1 mice still exhibiting control properties, while others exhibit AD-linked alterations. From this point of view, it would be useful to have a way to distinguish if a trace recorded in an APPPS1 mice belongs to a cell already affected by the ongoing pathology or not. For this reason, we performed a classification task using the 11 current-dependent features measured in this work. In Table 3, we report the best classification accuracy for each case. We first investigated the possibility to distinguish traces recorded from animal under control or AD; the *Ensemble of Bagged Trees* gave 75.1% classification accuracy for traces at 1 m, 73.3% at 4 m and 75.1% at 10 m. Next, we considered the possibility to identify traces affected by AD, after lumping together traces from all ages. We found that the classification accuracy was lower (69.5%), but still above chance level. This was somewhat expected since AD-1m were supposed to be very similar to control, and can thus reduce the likelihood to be distinguished from control. We also tested if it was possible to predict the age of an animal from a single trace. After training and validating on all the traces from control, using an *Ensemble Boosted Tree* classifier, we obtained an accuracy of 82.4%, which raised to 84.7% in predicting the age using all traces from the AD groups.

**TABLE 3:** Cluster analysis.

Group	Classification task	Classification accuracy	Classifier
*WT and AD at 1m*	WT or AD	75.1%	Ensemble Bagged Trees
*WT and AD at 4m*	WT or AD	73.3%	Ensemble Bagged Trees
*WT and AD at 10m*	WT or AD	75.1%	Ensemble Bagged Trees
*WT and AD at 1, 4, 10m*	WT or AD	69.5%	Ensemble Bagged Trees
*WT at 1, 4, 10m*	1m or 4m or 10m	82.4%	Ensemble Bagged Trees
*AD at 1, 4, 10m*	1m or 4m or 10m	84.7%	Ensemble Boosted Trees

*The columns represent the *Group* used for training and validation, the type of *Classification task* (i.e., assign a trace to one group), the probability of correct classification (*Classification accuracy*) and type of *Classifier* used for training.*

Taken together, these results suggest that it is possible to successfully classify a trace as belonging to an animal affected by AD, with an accuracy well above chance. Interestingly, the classification can also reliably predict the age of the animal from a single trace, suggesting that age itself is also an important determinant of the electrophysiological features exhibited by mouse hippocampal pyramidal neurons, both under control and AD conditions.

## Discussion

In this study, we took advantage of the new feature extraction tool of the HBP platform to systematically analyze 14 features of whole-cell patch clamp recordings of CA1 pyramidal neurons at three ages of APPPS1 mice, representing three stages of Aβ amyloidopathy, and compared these features to their respective WT control. One could also extract age-dependent alterations in these parameters using the WT recordings of our analysis. Our study was, however, not specifically designed for this type of evaluation. Indeed, recordings were interleaved between WT and APPPS1 mice within the same age group, usually on the same day of recording, allowing for accurate comparison of genotype effect per age group. To analyze age-dependent alterations in WT mice, we would have designed the study differently by interleaving recordings of different WT age groups within the same days. For this reason, we preferred not to analyze or discuss potential age-dependent alterations in neuron excitability measured in WT mice. Aging-related alterations in neuron excitability of the hippocampus have been investigated previously as reviewed in Oh et al. (2016). We report here strong alterations in membrane time constant and AP width, and weak alterations in *I*_*h*_ current and spiking behavior that correlate with different levels of amyloidopathy in this APPPS1 mouse model. Of note, the membrane time constant, the AP width and spike count exhibited an interesting phenotypic pattern in APPPS1 mice in that they reversed with increasing amyloidopathy. Membrane time constant and AP width were increased in amyloidopathy-free (1 months old) APPS1 mice, but then decreased in mice with weak and strong amyloidopathy. In the opposite way, spike count was decreased in amyloidopathy-free mice, but then increased in APPPS1 mice with strong amyloidopathy. Potential explanations for these findings will be discussed in detail below. We took advantage of these extracted features to perform a cluster analysis and we reliably (69–85%) identified traces to their respective group.

The time constant of the membrane is traditionally associated with the passive properties of a neuron, but its value can also be modulated by any active current that is significantly open around resting potential. Modulation of the membrane time constant, as a function of membrane voltage, can significantly alter the synaptic temporal integration window and the balance between excitation and inhibition, as a function of the synaptic activation frequency. This could at least be partly due to modulation of the hyperpolarization-activated cation current (*I*_*h*_), that contributes to the sag amplitude, and which is expressed at high density in CA1 pyramidal neurons playing a major role in modulating subthreshold signals (Magee, 1998). A progressive change in the *I*_*h*_ properties, in response to an increase in Aβ load, could explain the observed switch of the membrane time constant as mice get older. From this point of view, the *I*_*h*_ can play a predominant role. In our case, the model suggested that, keeping constant the mice age, the passive properties (*R*_*m*_ and *e__*pas*_*) did not change much under the control and AD conditions. However, the model supports the notion that amyloidopathy in 4-month animals seems to be a crucial step in the modification of neuronal activity, with some compensatory mechanism in effect. Indeed, while in control neurons, independently from the age and in AD-1m mice, the *I*_*h*_ peak conductance remains constant, its value increases by 50% (increasing the current) with amyloidopathy in AD-4m and AD-10m (Table 2) but, at the same time, the activation is modified in such a way to reduce the overall current.

The main difference observed for AP waveform analysis is the alterations in AP width. The narrowing of this width (∼10–15%) with progression of amyloidopathy is one of the unique features that has been consistently observed in CA1 pyramidal neurons in other AD mouse models at ages representing weak and strong amyloidopathy, such as in the PDAPP mice (Kerrigan et al., 2014; Tamagnini et al., 2015), in the PSAPP mice (Brown et al., 2011) and in the CRND8 mice (Wykes et al., 2012). This narrower AP width could stem from faster sodium channel inactivation, alterations in fast gating potassium channels with roles in AP repolarization, or loss of a contribution from voltage-gated calcium channels to AP waveform. Wykes et al. (2012) proposed the increased expression in the potassium channel Kv3.1b as a potential mechanism in the CRND8 mouse model. There is also accumulating evidence that *in vitro* and *in vivo* exposure of hippocampal neurons to Aβ modifies voltage-gated sodium, potassium and calcium channels (Pannaccione et al., 2007; Durán-González et al., 2013; Mayordomo-Cava et al., 2015; Gavello et al., 2018; Ciccone et al., 2019; Wang et al., 2019). We can thus reasonably suggest that this consistent phenotype observed in the different AD mouse models is likely to be due to Aβ accumulation and relevant to the human AD pathology.

Surprisingly, when we analyzed the APPPS1 mice at a very young age when no detectable increase in Aβ levels in the brain were reported (Radde et al., 2006; Maia et al., 2013), we observed wider AP width, a complete opposite phenotype to the one observed with weak and strong amyloidopathy. This very early phenotype was not previously observed in other studies, but recording of other mouse models at this very young amyloidopathy-free age has not yet been reported. This phenotype could be due to the early effect of mutated APP and PS1 transgene expressions *per se*. As mentioned above, voltage-gated sodium, potassium, and calcium channels govern AP width. A first analysis of voltage-gated sodium channel kinetics with the phase-plots did not show alterations in these channels at any ages in these mice. Our recordings did not allow us to evaluate potential alterations in voltage-gated potassium or calcium channels. However, there is evidence that overexpression of APP *per se* increases sodium channel currents and cell surface expression (Liu et al., 2015; Li et al., 2016). Also, the voltage-gated sodium channel beta2-subunit, an auxiliary subunit of the voltage-gated sodium channel involved both in channel gating and cell surface expression of α subunits of the sodium channel, was shown to be a substrate for PS1-dependent gamma-secretase-mediated cleavage (Kim et al., 2005). Furthermore, overexpression of a mutated PS1 (albeit the deltaE9 mutation) decreased potassium channel current and cell surface expression (Plant et al., 2002). It is therefore reasonable to speculate that overexpression of mutated APP and PS1 levels could significantly alter AP kinetics by acting on sodium and potassium channels. Independent of Aβ accumulation, these types of alterations could contribute to the widening of the AP width observed in very young APPPS1 mice. With age, the brain of these mice might be slowly adapting to cellular alterations provoked by this overexpression within the first months. With progressive amyloidopathy, AP width then reverses phenotype, becoming abnormally narrower as observed in the other AD mouse models with ongoing amyloidopathy. Together these data suggest that AP width alterations are closely linked to APP processing and the underlying molecular mechanisms should be further investigated to identify this relationship.

Spike count was decreased in young amyloidopathy-free mice, suggesting hypoexcitability, and increased in old APPPS1 mice with strong amyloidopathy, suggesting hyperexcitability. These phenotypes were however only observed at high current steps (350 and 400 pA) and not at lower current steps (50–300 pA), which are likely to represent more physiological conditions. Regarding these findings in strong amyloidopathy conditions, our data confirm other studies using a similar protocol with the lack of phenotype at weak current injections (100–300 pA) as observed in the PDAPP model (Kerrigan et al., 2014; Tamagnini et al., 2015). They however do not support a study in PSAPP mice where hypoexcitability was observed (Brown et al., 2011). Yet, all three previous studies identified higher instantaneous frequency with weak and strong amyloidopathy in these other two models (Brown et al., 2011; Kerrigan et al., 2014; Tamagnini et al., 2015). Here, we report lower instantaneous frequency in 9–10 months old APPPS1 mice. At present, we cannot explain this discrepancy. Nevertheless, altogether these data suggest that CA1 pyramidal neurons exposed to strong amyloidopathy are quite resistant to disruption in firing behavior.

Surprisingly, in our model, both spike count and instantaneous frequency measures transiently normalized in 3–4 months old mice exhibiting weak amyloidopathy. We could speculate that in this APPPS1 model early overexpression of mutated APP and PS1 perturb homeostatic processes that drive neuron excitability and that the brain transiently copes with these alterations restoring the required homeostatic balance. With aging and increasing amyloidopathy, the network cannot cope with the increasing cellular modifications slowly degrading homeostatic processes with increasing perturbations of firing behavior. We currently have not proof of this possibility in this model, but the general concept of loss of firing homeostasis in the context of AD has been well described in a recent review (Styr and Slutsky, 2018).

Previous studies reporting the activity profile of CA1 pyramidal neurons *in vivo* in models similar to our APPPS1 model, i.e., overexpressing mutated forms of APP and of PS1, support our data correlating high levels of Aβ-amyloidopathy, including Aβ plaques, with increased excitability. Indeed, female and male mice (10–14 months) heterozygous for both transgenes, K670N/M671L-mutated amyloid precursor protein (APPswe), and the M146V mutated presenilin 1 (PS1) under control of a neuron-specific promoter (Willuweit et al., 2009), which display strong Aβ deposition, exhibited increased action potential frequency in CA1 pyramidal neurons *in vivo* (Šišková et al., 2014). These authors analyzed these mice at 2–4 months, before Aβ plaque appearance, and did not observe alterations in CA1 firing behavior *in vivo* at this age. However, we do not know the levels of Aβ accumulation in 2–4 months old mice of this AD model, and these could represent early Aβ accumulation with rise in soluble forms of Aβ, similar to our 3–4 months APPPS1 mice where we also did not evidence any alterations in firing behavior. Furthermore, another similar mouse model, the APP23xPS45 double transgenic mouse model overexpressing the human APP with the Swedish (670/671) mutation and the human G384A-mutated presenilin 1 (PS1) (Busche et al., 2008) was analyzed for alterations in spontaneous calcium transients *in vivo* as a measure of neuron activity in the CA1 region of the hippocampus (Busche et al., 2012). In this study, they report that Aβ accumulation, before plaques and after plaque deposition, correlates with increased hyperactivity of CA1 pyramidal neurons. This study did not analyze AD mice before an increase of soluble Aβ. They also report that an acute application of soluble synthetic AβS26C dimer (100 nM) itself is sufficient to increase CA1 pyramidal neuron hyperactivity in WT mice. Together these data confirm our finding that a strong increase in Aβ load correlates with hyperactivity of CA1 pyramidal neurons.

To summarize our findings, we noted strong age-dependent alterations in membrane time constant and AP width and weak age-dependent alterations in *I*_*h*_ current and firing behavior correlating with levels of Aβ amyloidopathy. It is important to note that we have no evidence that Aβ accumulation is mediating these phenotypes. Unfortunately, using these types of APP and PS1 overexpressing AD models, it will be difficult to sort out what phenotype is directly due to Aβ accumulation, and what phenotype is due to overexpression of mutated APP or PS1 as detailed above, or to accumulation of other cleaved APP peptides. For example, the intracellular C-terminal domain (AICD) of APP is also overproduced in these APP-based AD models (Pousinha et al., 2017) and there is evidence that it can also modulate *per se* CA1 pyramidal neuron excitability (Pousinha et al., 2019). A throughout evaluation of a new type of AD mouse models exhibiting progressive amyloidopathy, the APP knock-in models (Saito et al., 2014), could be useful to address some of these issues. Indeed, in this model of Aβ amyloidopathy, which progresses at a much slower rate than in APP over-expressing models, the endogenous mouse APP gene was replaced by a humanized form of the APP gene containing familial AD mutations. The human APP gene is thus driven by the endogenous mouse APP promoter avoiding APP overexpression.

One of the hallmarks of AD is the formation of more or less spatially sparse and distributed plaques that progressively hinder neuronal functions. This implies that, at any given time, there can be a significant population of still “normal” neurons in an APPPS1 animal. One limitation of our work is that, in old APPS1 mice, that harbor Aβ plaques, we could not directly relate the location of the patched neurons to the vicinity of these plaques. It is possible that, under this condition, we have patched neurons that were differently affected due to their location with respect to these plaques. Yet, the clustering analysis point out to an interesting possibility. This approach may help in preferentially selecting for analysis only those neurons that can be classified as affected by amyloidopathy, reducing the overall variability in the measured differences between control and AD animals.

## Conclusion

We identified specific features of neuron excitability that correlated best with either with over-expression of mutated APP and PS1 (in AD-1m mice) or increasing Aβ amyloidopathy (in AD-4m and AD-10m mice). By improving our knowledge of the ionic mechanisms that are most affected by AD, and by making experimentally testable predictions on the role of *I*_*h*_, the results can be usefully exploited by both modelers and experimentalists. Modelers can use them for computational modeling studies on the hippocampal network under AD conditions, and experimentalists can use our clustering analysis to improve the quality of their analyses on the effects of amyloidopathy.

## Data Availability Statement

The datasets presented in this study can be found in online repositories. The names of the repository/repositories and accession number(s) can be found below: data and models are available at the live paper section of ebrains (https://humanbrainproject.github.io/hbp-bsp-live-papers/2021/vitale_et_al_2021/vitale_et_al_2021.html). Model is also available on Model DB accession no. 266848 (https://senselab.med.yale.edu/ModelDB/showmodel.cshtml?model=266848#tabs-1).

## Ethics Statement

The animal study was reviewed and approved by the French Research Ministry.

## Author Contributions

AS-P and HM maintained the mouse colony, performed the genotyping, and performed the electrophysiology experiments. PV and RM analyzed the data. CL performed the cluster analysis. MW performed the biochemical analysis of Aβ load. HM and MM designed the study and wrote manuscript with help from all other authors. All authors contributed to the article and approved the submitted version.

## Conflict of Interest

The authors declare that the research was conducted in the absence of any commercial or financial relationships that could be construed as a potential conflict of interest.

## Footnotes

1 https://www.alzforum.org/research-models/appps1

2 https://www.humanbrainproject.eu/en/hbp-platforms

3 https://github.com/BlueBrain/eFEL

4 https://senselab.med.yale.edu/modeldb/

5 https://humanbrainproject.github.io/hbp-bsp-live-papers/2018/migliore_et_al_2018/migliore_et_al_2018.html
