# Supplementary material for: Analysis of Age-Dependent Alterations in Excitability Properties of CA1 Pyramidal Neurons in an APPPS1 Model of Alzheimer’s Disease
Source: Front Aging Neurosci. 2021 Jun 11;13:668948. doi: 10.3389/fnagi.2021.668948 (PMC8230571; doi:10.3389/fnagi.2021.668948)
Supplement: Supplementary file 1 [file Table_1.DOCX]

Vitale et al. - Supplementary Material

Table S1: ELISA assay


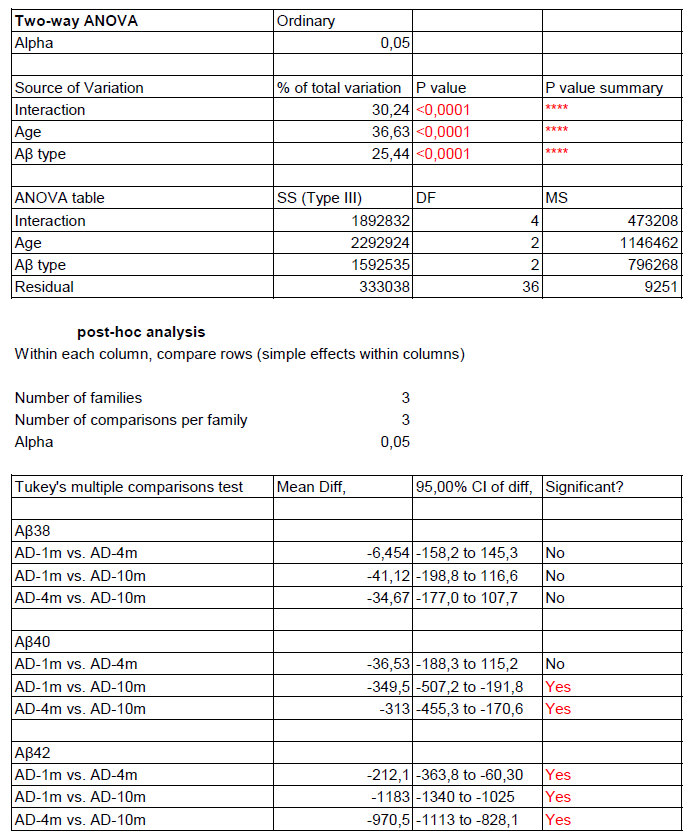


Table S2: Passive membrane properties

| **Features name** | **Current**  **(pA)** | **WT-1m**  **n = 14** | **AD-1m**  **n = 11** | ***p*** | **WT-4m**  **n = 12** | **AD-4m**  **n = 15** | ***p*** | **WT-10m**  **n = 19** | **AD-10m**  **n = 21** | ***p*** |
| --- | --- | --- | --- | --- | --- | --- | --- | --- | --- | --- |
| **Input resistance**  **(GΩ)** | - | 169 ± 16 | 129 ± 19 | 0.133 | 131 ± 25 | 166 ± 15 | 0.207 | 132 ± 15 | 114 ± 17 | 0.369 |
| **Resting Membrane**  **Potential (mV)** | - | -64.2 ± 0.5 | -63.3 ± 0.6 | 0.291 | -66.7 ± 1.4 | -65.5 ± 1.2 | 0.516 | -64.3 ± 0.9 | -66.2 ± 0.8 | 0.060 |
| **Voltage_base**  **(mV)** | -200  -150  -100  -50  50  100  150  200  250  300  350  400 | -79.0 ± 0.2  -79.1 ± 0.2  -78.9 ± 0.2  -78.6 ± 0.3  -78.6 ± 0.2  -79.1 ± 0.3  -79.6 ± 0.4  -80.2 ± 0.4  -80.6 ± 0.4  -80.7 ± 0.4  -81.0 ± 0.4  -81.3 ± 0.5 | -79.1 ± 0.1  -78.9 ± 0.2  -79.0 ± 0.2  -78.7 ± 0.2  -78.7 ± 0.3  -78.8 ± 0.3  -79.0 ± 0.3  -79.4 ± 0.3  -79.7 ± 0.3  -79.9 ± 0.4  -80.2 ± 0.3  -80.2 ± 0.4 | 0.742  0.261  0.068  0.448  0.925  0.316  0.247  0.103  0.084  0.118  0.116  0.136 | -80.8 ± 0.5  -80.8 ± 0.5  -80.5 ± 0.4  **-80.3 ± 0.6**  **-79.8 ± 0.6**  -79.7 ± 0.7  -80.3 ± 0.6  -80.9 ± 0.8  -81.7 ± 0.6  -81.9 ± 0.4  -82.2 ± 0.3  -81.7 ± 0.6 | -81.4 ± 0.4  -81.7 ± 0.4  -81.5 ± 0.4  **-81.7 ± 0.4**  **-81.5 ± 0.5**  -81.4 ± 0.6  -81.8 ± 0.5  -82.0 ± 0.7  -82.3 ± 0.6  -82.3 ± 0.7  -82.7 ± 0.7  -82.4 ± 0.8 | 0.315  0.160  0.122  **0.050**  **0.050**  0.070  0.063  0.129  0.497  0.608  0.491  0.506 | -79.7 ± 0.2  -79.7 ± 0.2  -79.8 ± 0.3  -79.8 ± 0.3  -79.7 ± 0.3  -79.6 ± 0.3  -80.0 ± 0.4  -80.1 ± 0.4  -80.5 ± 0.4  -80.6 ± 0.4  -80.7 ± 0.4  -80.9 ± 0.4 | -79.5 ± 0.4  -79.8 ± 0.4  -79.9 ± 0.5  -79.7 ± 0.5  -79.7 ± 0.6  -79.9 ± 0.5  -80.1 ± 0.5  -80.3 ± 0.5  -80.4 ± 0.5  -80.5 ± 0.4  -80.3 ± 0.4  -80.4 ± 0.4 | 0.712  0.896  0.874  0.897  0.551  0.656  0.807  0.719  0.862  0.806  0.452  0.308 |
| **Steady_state_Voltage**  **(mV)** | -200  -150  -100  -50  50  100  150  200  250  300  350  400 | -78.7 ± 0.1  -78.8 ± 0.2  -78.7 ± 0.2  -78.7 ± 0.3  -79.1 ± 0.3  -79.7 ± 0.3  -80.4 ± 0.4  -81.0 ± 0.4  -81.5 ± 0.5  -81.7 ± 0.5  -82.0 ± 0.5  -82.1 ± 0.5 | -78.9 ± 0.2  -78.9 ± 0.2  -78.7 ± 0.2  -78.6 ± 0.2  -78.7 ± 0.2  -79.1 ± 0.3  -79.5 ± 0.3  -80.0 ± 0.3  -80.4 ± 0.4  -80.7 ± 0.4  -80.9 ± 0.4  -81.1 ± 0.5 | 0.414  0.888  0.932  0.820  0.321  0.221  0.107  0.103  0.084  0.118  0.116  0.136 | -80.5 ± 0.4  -80.4 ± 0.4  **-79.8 ± 0.5**  -80.2 ± 0.5  -79.6 ± 0.8  -80.3 ± 0.6  -81.0 ± 0.7  -81.3 ± 0.7  -81.9 ± 0.5  -82.1 ± 0.5  -82.3 ± 0.4  -82.2 ± 0.5 | -81.4 ± 0.4  -81.5 ± 0.4  **-81.5 ± 0.4**  -81.4 ± 0.5  -81.3 ± 0.5  -81.8 ± 0.5  -82.2 ± 0.5  -82.5 ± 0.6  -82.7 ± 0.7  -82.8 ± 0.7  -83.3 ± 0.7  -83.2 ± 0.8 | 0.143  0.097  **0.017**  0.097  0.080  0.083  0.102  0.143  0.180  0.459  0.158  0.231 | -79.6 ± 0.2  -79.7 ± 0.3  -79.7 ± 0.3  -79.5 ± 0.3  -79.6 ± 0.3  -79.8 ± 0.3  -80.2 ± 0.3  -80.6 ± 0.4  -81.0 ± 0.4  -81.2 ± 0.4  -81.2 ± 0.4  -81.6 ± 0.4 | -79.2 ± 0.4  -79.5 ± 0.5  -79.5 ± 0.6  -79.4 ± 0.6  -79.6 ± 0.5  -80.1 ± 0.5  -80.7 ± 0.5  -80.9 ± 0.4  -81.3 ± 0.4  -81.2 ± 0.4  -81.1 ± 0.4  -81.2 ± 0.4 | 0.149  0.537  0.477  0.414  0.491  0.663  0.474  0.354  0.682  0.904  0.867  0.464 |
| **Time constant**  **(ms)** | -200  -150  -100  -50 | **12.8 ± 0.5**  **13.3 ± 0.6**  **14.8 ± 0.5**  **16.4 ± 1.0** | **15.8 ± 1.0**  **17.5 ± 1.2**  **18.5 ± 1.0**  **20.1 ± 1.4** | **0.034**  **0.004**  **0.009**  **0.039** | 11.9 ± 0.5  12.8 ± 0.5  14.4 ± 0.3  **17.4 ± 0.8** | 10.9 ± 0.4  11.7 ± 0.5  13.3 ± 0.5  **14.1 ± 0.7** | 0.052  0.126  0.479  **0.006** | **13.4 ± 0.6**  **14.7 ± 0.7**  **15.9 ± 0.9**  **17.8 ± 0.9** | **11.3 ± 0.4**  **12.2 ± 0.4**  **13.4 ± 0.4**  **15.3 ± 0.6** | **0.010**  **0.004**  **0.048**  **0.041** |
| **Sag amplitude**  **(mV)** | -200  -150  -100  -50 | 5.4 ± 0.4  4.8 ± 0.4  3.7 ± 0.4  1.8 ± 0.3 | 6.5 ± 0.4  5.4 ± 0.4  4.1 ± 0.3  2.3 ± 0.2 | 0.060  0.337  0.158  0.095 | 5.2 ± 0.3  3.8 ± 0.2  2.8 ± 0.2  1.6 ± 0.2 | 4.8 ± 0.3  4.1 ± 0.3  2.7 ± 0.2  1.5 ± 0.1 | 0.397  0.436  0.643  0.805 | **5.6 ± 0.3**  4.5 ± 0.3  3.2 ± 0.2  **1.7 ± 0.1** | **4.5 ± 0.3**  3.8 ± 0.2  2.8 ± 0.1  **1.4 ± 0.1** | **0.034**  0.103  0.111  **0.013** |
| **Minimum voltage**  **Between spikes**  **(mV)** | 50  100  150  200  250  300  350  400 | -70.9 ± 1.2  -65.8 ± 1.1  -63.1 ± 1.2  -60.8 ± 1.3  -58.8 ± 1.4  -56.8 ± 1.5  -55.0 ± 1.6  -53.3 ± 1.7 | -69.3 ± 1.1  -63.2 ± 1.2  -60.3 ± 1.4  -58.1 ± 1.5  -56.0 ± 1.6  -54.2 ± 1.7  -52.5 ± 1.9  -50.7 ± 1.9 | 0.342  0.109  0.138  0.182  0.210  0.260  0.318  0.314 | -74.1 ± 1.1  -70.1 ± 1.1  -67.6 ± 1.2  -65.0 ± 1.2  -62.3 ± 1.2  -59.9 ± 1.4  -57.7 ± 1.7  -55.6 ± 1.8 | -73.8 ± 1.6  -71.5 ± 1.4  -68.9 ± 1.2  -66.1 ± 1.3  -63.7 ± 1.3  -61.5 ± 1.3  -59.4 ± 1.5  -57.5 ± 1.8 | 0.966  0.221  0.294  0.285  0.473  0.416  0.503  0.600 | -68.7 ± 1.5  -66.2 ± 1.4  -63.4 ± 1.6  -60.5 ± 1.8  -58.0 ± 2.0  -55.7 ± 2.1  -53.9 ± 2.3  -51.9 ± 2.5 | -71.1 ± 1.5  -66.4 ± 0.7  -63.0 ± 0.8  -60.0 ± 0.9  -57.1 ± 1.2  -55.5 ± 1.0  -52.8 ± 1.1  -50.7 ± 1.2 | 0.305  0.866  0.860  0.930  0.792  0.939  0.562  0.662 |

**Table S3: First AP features**

| **Features name** | **Current (pA)** | **WT-1m**  **n = 14** | **AD-1m**  **n = 11** | ***p*** | **WT-4m**  **n = 12** | **AD-4m**  **n = 15** | ***p*** | **WT-10m**  **n = 19** | **AD-10m**  **n = 21** | ***p*** |
| --- | --- | --- | --- | --- | --- | --- | --- | --- | --- | --- |
| **Time to**  **first spike**  **(ms)** | 50  100  150  200  250  300  350  400 | 35.0 ± 8.2  27.6 ± 4.9  14.4 ± 2.0  9.4 ± 1.0  6.9 ± 0.6  5.4 ± 0.5  4.4 ± 0.4  3.8 ± 0.3 | 46.1 ± 7.8  24.6 ± 3.6  12.6 ± 1.7  8.6 ± 1.2  6.6 ± 0.8  5.3 ± 0.6  4.4 ± 0.5  3.8 ± 0.4 | 0.138  0.637  0.496  0.604  0.742  0.813  0.978  0.970 | **31.4 ± 11.6**  11.4 ± 2.4  6.9 ± 1.5  4.8 ± 0.8  3.6 ± 0.6  3.2 ± 0.4  3.0 ± 0.4  2.5 ± 0.3 | **99.1 ± 26.1**  13.3 ± 1.4  9.3 ± 1.4  6.9 ± 1.3  5.6 ± 1.4  5.0 ± 1.6  4.6 ± 1.7  4.5 ± 2.0 | **0.015**  0.353  0.180  0.127  0.063  0.241  0.787  0.857 | 86.2 ± 29.9  25.7 ± 2.5  13.1 ± 1.0  8.6 ± 0.8  6.4 ± 0.5  5.1 ± 0.4  4.4 ± 0.4  3.8 ± 0.3 | 50.4 ± 22.2  27.4 ± 4.5  11.6 ± 1.3  7.6 ± 0.7  5.5 ± 0.5  4.5 ± 0.4  4.0 ± 0.3  3.4 ± 0.2 | 0.072  0.607  0.400  0.291  0.209  0.286  0.392  0.290 |
| **frequency**  **of first ISI**  **(Hz)** | 50  100  150  200  250  300  350  400 | 30.3 ± 5.5  45.8 ± 6.8  75.3 ± 6.3  94.3 ± 6.0  109.5 ± 5.6  127.0 ± 5.9  141.0 ± 6.0  152.2 ± 5.7 | 40.7 ± 13.2  57.2 ± 8.7  84.6 ± 6.5  102.0 ± 6.9  121.3 ± 6.5  132.8 ± 6.2  145.6 ± 6.1  154.6 ± 5.4 | 0.456  0.306  0.328  0.406  0.184  0.517  0.610  0.848 | 22.8 ± 3.7  47.7 ± 4.8  68.9 ± 6.0  90.8 ± 6.6  109.0 ± 5.6  125.2 ± 6.2  139.8 ± 6.1  149.5 ± 4.1 | 8.6 ± 1.6  42.8 ± 5.8  57.8 ± 8.5  78.4 ± 8.9  97.3 ± 10.1  113.1 ± 11.4  132.7 ± 8.6  143.2 ± 10.5 | 0.066  0.568  0.525  0.228  0.264  0.320  0.552  0.441 | 19.5 ± 7.5  48.2 ± 7.6  72.4 ± 8.6  89.7 ± 9.2  105.6 ± 9.7  **118.5 ± 9.7**  **125.4 ± 10.6**  **134.8 ± 10.2** | 21.3 ± 3.4  48.0 ± 5.8  84.0 ± 6.4  109.5 ± 6.0  129.2 ± 5.6  **145.6 ± 5.7**  **158.5 ± 6.2**  **172.1 ± 5.7** | 0.804  0.988  0.138  0.159  0.069  **0.043**  **0.019**  **0.001** |
| **First**  **AP width**  **(ms)** | 50  100  150  200  250  300  350  400 | **0.86 ± 0.04**  **0.91 ± 0.03**  **0.94 ± 0.04**  **0.91 ± 0.03**  **0.93 ± 0.03**  **0.92 ± 0.03**  **0.94 ± 0.03**  **0.97 ± 0.03** | **1.02 ± 0.03**  **1.03 ± 0.03**  **1.04 ± 0.02**  **1.04 ± 0.02**  **1.06 ± 0.02**  **1.06 ± 0.03**  **1.08 ± 0.03**  **1.10 ± 0.03** | **0.016**  **0.022**  **0.026**  **0.006**  **0.004**  **0.002**  **0.004**  **0.012** | 0.99 ± 0.04  **1.02 ± 0.02**  **1.00 ± 0.02**  **0.99 ± 0.03**  **1.02 ± 0.02**  **1.02 ± 0.02**  **1.06 ± 0.03**  1.05 ± 0.03 | 0.91 ± 0.02  **0.89 ± 0.02**  **0.89 ± 0.03**  **0.89 ± 0.03**  **0.89 ± 0.02**  **0.93 ± 0.03**  **0.97 ± 0.03**  0.99 ± 0.03 | 0.089  **0.003**  **0.017**  **0.010**  **0.001**  **0.016**  **0.032**  0.208 | 0.97 ± 0.04  0.96 ± 0.02  0.97 ± 0.02  **0.99 ± 0.02**  **1.02 ± 0.01**  **1.06 ± 0.02**  1.07 ± 0.03  **1.09 ± 0.04** | 0.85 ± 0.06  0.91 ± 0.03  0.91 ± 0.03  **0.92 ± 0.03**  **0.95 ± 0.03**  **0.95 ± 0.04**  0.99 ± 0.04  **0.99 ± 0.04** | 0.106  0.117  0.105  **0.050**  **0.033**  **0.021**  0.063  **0.046** |
| **First AP**  **amplitude**  **(mV)** | 50  100  150  200  250  300  350  400 | 84.3 ± 3.3  81.6 ± 2.4  81.7 ± 2.3  82.1 ± 2.3  82.3 ± 2.5  84.2 ± 2.6  88.8 ± 2.7  93.4 ± 1.6 | 90.1 ± 3.2  84.6 ± 2.5  84.8 ± 2.6  84.9 ± 2.6  85.0 ± 2.5  84.4 ± 2.6  95.0 ± 2.3  95.9 ± 2.3 | 0.235  0.389  0.384  0.428  0.455  0.974  0.105  0.208 | 95.9 ± 2.1  95.8 ± 1.7  96.1 ± 1.7  96.1 ± 2.0  96.1 ± 2.0  95.5 ± 2.2  99.1 ± 0.9  98.2 ± 1.4 | 94.4 ± 2.6  94.5 ± 2.5  93.1 ± 2.5  93.0 ± 2.7  93.0 ± 2.8  95.3 ± 2.4  98.4 ± 1.1  99.3 ± 1.5 | 0.972  0.772  0.358  0.664  0.828  0.947  0.676  0.616 | 81.8 ± 5.5  83.6 ± 3.4  83.6 ± 3.4  84.1 ± 3.4  88.2 ± 2.6  93.0 ± 2.5  **97.3 ± 2.2**  98.1 ± 2.1 | 79.1 ± 4.7  82.1 ± 2.7  81.7 ± 2.7  81.5 ± 2.8  83.8 ± 2.7  88.1 ± 1.7  **89.5 ± 2.0**  92.1 ± 2.0 | 0.720  0.733  0.673  0.550  0.335  0.103  **0.013**  0.055 |

**Table S4: Intrinsic neuron excitability**

| **Features name** | **Current**  **(pA)** | **WT-1m**  **n = 14** | **AD-1m**  **n = 11** | ***p*** | **WT-4m**  **n = 12** | **AD-4m**  **n = 15** | ***p*** | **WT-10m**  **n = 19** | **AD-10m**  **n = 21** | ***p*** |
| --- | --- | --- | --- | --- | --- | --- | --- | --- | --- | --- |
| **Spike count**  **(n)** | 50  100  150  200  250  300  350  400 | 3.2 ± 1.4  10.6 ± 1.4  16.6 ± 1.1  20.8 ± 1.0  23.4 ± 0.9  25.3 ± 1.0  **26.9 ± 1.0**  **28.3 ± 1.0** | 3.6 ± 1.1  11.5 ± 1.1  16.4 ± 0.9  19.3 ± 0.8  21.4 ± 0.9  22.8 ± 0.8  **24.1 ± 0.8**  **24.9 ± 0.8** | 0.794  0.631  0.934  0.287  0.129  0.063  **0.046**  **0.019** | **5.5 ± 0.6**  11.2 ± 0.8  15.3 ± 0.9  18.3 ± 0.9  20.6 ± 0.8  22.2 ± 0.7  23.4 ± 0.8  24.1 ± 0.9 | **2.0 ± 0.4**  9.2 ± 1.1  14.1 ± 1.3  17.6 ± 1.3  20.1 ± 1.4  21.9 ± 1.4  24.3 ± 1.0  25.2 ± 1.0 | **< 0.001**  0.238  0.361  0.533  0.638  0.713  0.325  0.591 | 1.5 ± 0.5  8.1 ± 0.8  13.3 ± 0.8  16.4 ± 0.8  18.7 ± 0.8  20.3 ± 0.8  **21.5 ± 0.9**  **22.5 ± 0.9** | 1.7 ± 0.6  8.7 ± 0.8  14.0 ± 0.7  17.6 ± 0.7  20.6 ± 0.7  22.3 ± 0.7  **24.0 ± 0.7**  **25.2 ± 0.7** | 0.852  0.635  0.520  0.297  0.229  0.077  **0.015**  **0.031** |
| **Mean**  **frequency**  **(Hz)** | 50  100  150  200  250  300  350  400 | 20.3 ± 3.5  28.3 ± 3.5  43.8 ± 2.8  53.3 ± 2.4  60.1 ± 2.4  65.2 ± 2.4  **69.2 ± 2.5**  **72.2 ± 2.4** | 18.1 ± 2.2  30.3 ± 2.7  42.6 ± 2.3  50.2 ± 2.1  55.3 ± 2.0  59.3 ± 2.1  **61.9 ± 2.0**  **64.3 ± 2.0** | 0.594  0.685  0.766  0.370  0.153  0.080  **0.037**  **0.025** | **16.7 ± 1.6**  30.4 ± 2.0  40.2 ± 2.0  47.5 ± 1.9  53.8 ± 1.8  57.3 ± 1.8  59.8 ± 2.2  62.0 ± 2.2 | **8.4 ± 1.1**  26.4 ± 2.1  38.7 ± 2.3  46.8 ± 2.8  53.8 ± 2.8  58.5 ± 2.7  61.9 ± 2.6  64.9 ± 3.0 | **< 0.001**  0.227  0.625  0.840  0.500  0.718  0.577  0.484 | 10.7 ± 2.1  24.6 ± 1.8  37.1 ± 1.7  45.2 ± 1.6  51.0 ± 1.5  55.0 ± 1.4  58.1 ± 1.7  60.3 ± 1.7 | 17.3 ± 2.4  24.0 ± 1.9  36.2 ± 1.9  45.8 ± 1.8  52.1 ± 1.7  57.4 ± 1.7  62.1 ± 1.8  64.8 ± 1.9 | 0.064  0.826  0.748  0.792  0.651  0.306  0.115  0.099 |
|  | **ISI number** |  |  |  |  |  |  |  |  |  |
| **Instantaneous**  **Frequency**  **at 300 pA**  **(Hz)** | 1  2  3  4  5  6  7  8  9  10 | 96.5 ± 3.7  85.8 ± 2.8  79.4 ± 2.8  75.3 ± 2.6  73.6 ± 2.6  72.3 ± 2.5  **71.1 ± 3.0**  68.6 ± 2.4  **68.4 ± 2.4**  **66.7 ± 2.8** | 97.9 ± 5.4  83.6 ± 4.4  75.4 ± 3.7  72.0 ± 2.9  67.8 ± 3.0  65.8 ± 2.9  **62.7 ± 2.4**  60.9 ± 3.6  **59.5 ± 3.0**  **57.4 ± 2.4** | 0.833  0.677  0.385  0.420  0.157  0.109  **0.044**  0.086  **0.027**  **0.011** | 93.3 ± 5.5  81.0 ± 4.3  70.1 ± 3.9  68.9 ± 3.4  65.9 ± 3.9  61.8 ± 3.2  59.1 ± 2.4  59.1 ± 3.3  57.0 ± 2.8  53.3 ± 2.3 | 88.9 ± 7.1  79.0 ± 6.2  72.9 ± 4.5  68.2 ± 4.3  66.3 ± 4.2  61.1 ± 3.8  61.8 ± 3.9  60.2 ± 3.4  59.4 ± 3.3  56.7 ± 3.3 | 0.633  0.799  0.649  0.447  0.939  0.890  0.588  0.818  0.593  0.425 | 90.2 ± 6.7  **76.8 ± 5.5**  **67.1 ± 4.1**  60.7 ± 3.2  **59.1 ± 3.1**  **56.6 ± 3.0**  **53.8 ± 2.9**  **52.0 ± 2.7**  **51.4 ± 2.8**  **51.5 ± 2.8** | 76.4 ± 4.7  **63.1 ± 4.1**  **55.0 ± 3.4**  53.1 ± 3.3  **50.2 ± 2.8**  **47.1 ± 2.5**  **43.6 ± 2.7**  **43.1 ± 2.6**  **41.5 ± 2.8**  **41.2 ± 2.4** | 0.092  **0.049**  **0.027**  0.053  **0.028**  **0.018**  **0.015**  **0.023**  **0.018**  **0.008** |

**A**

**B**


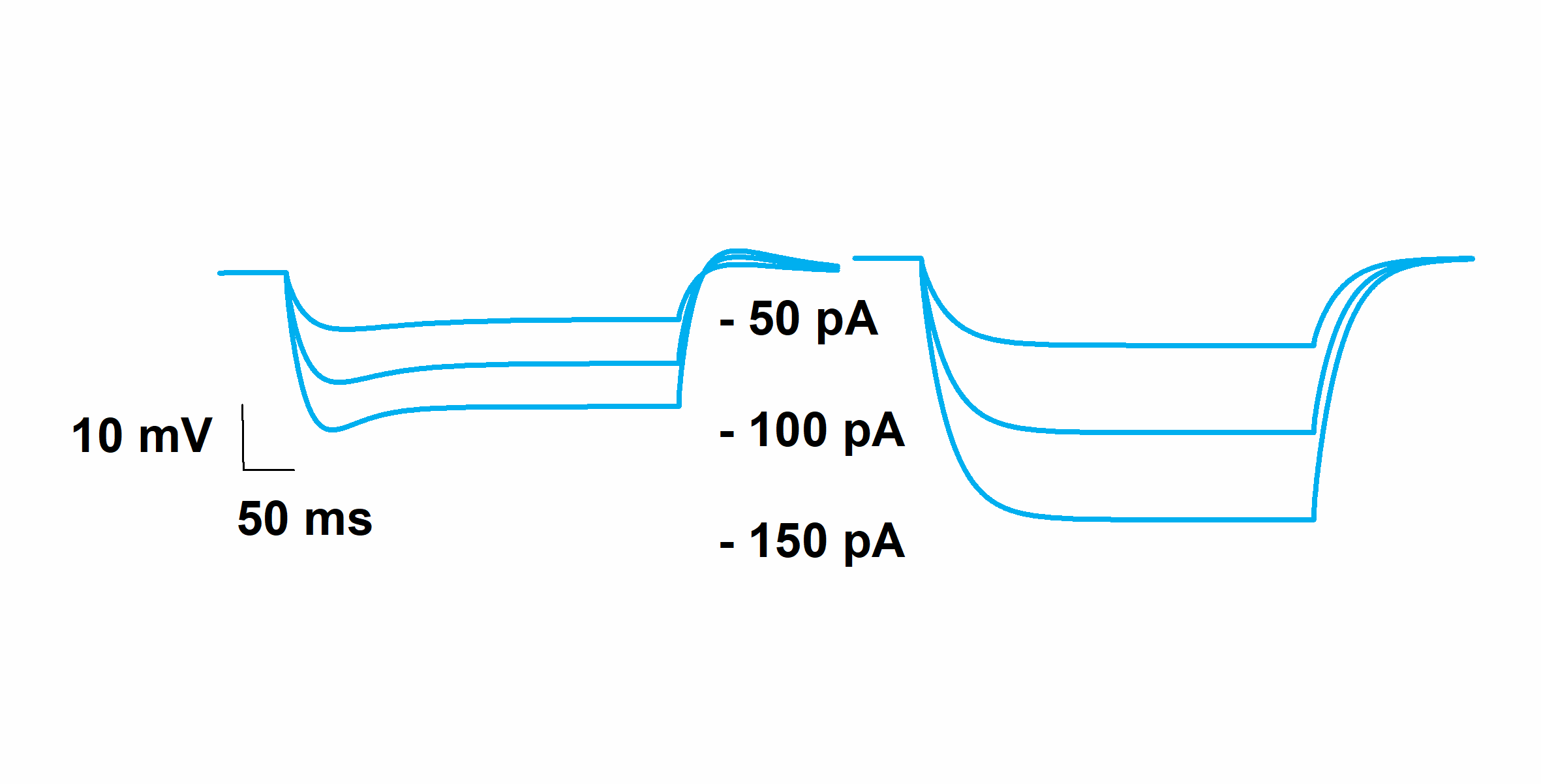

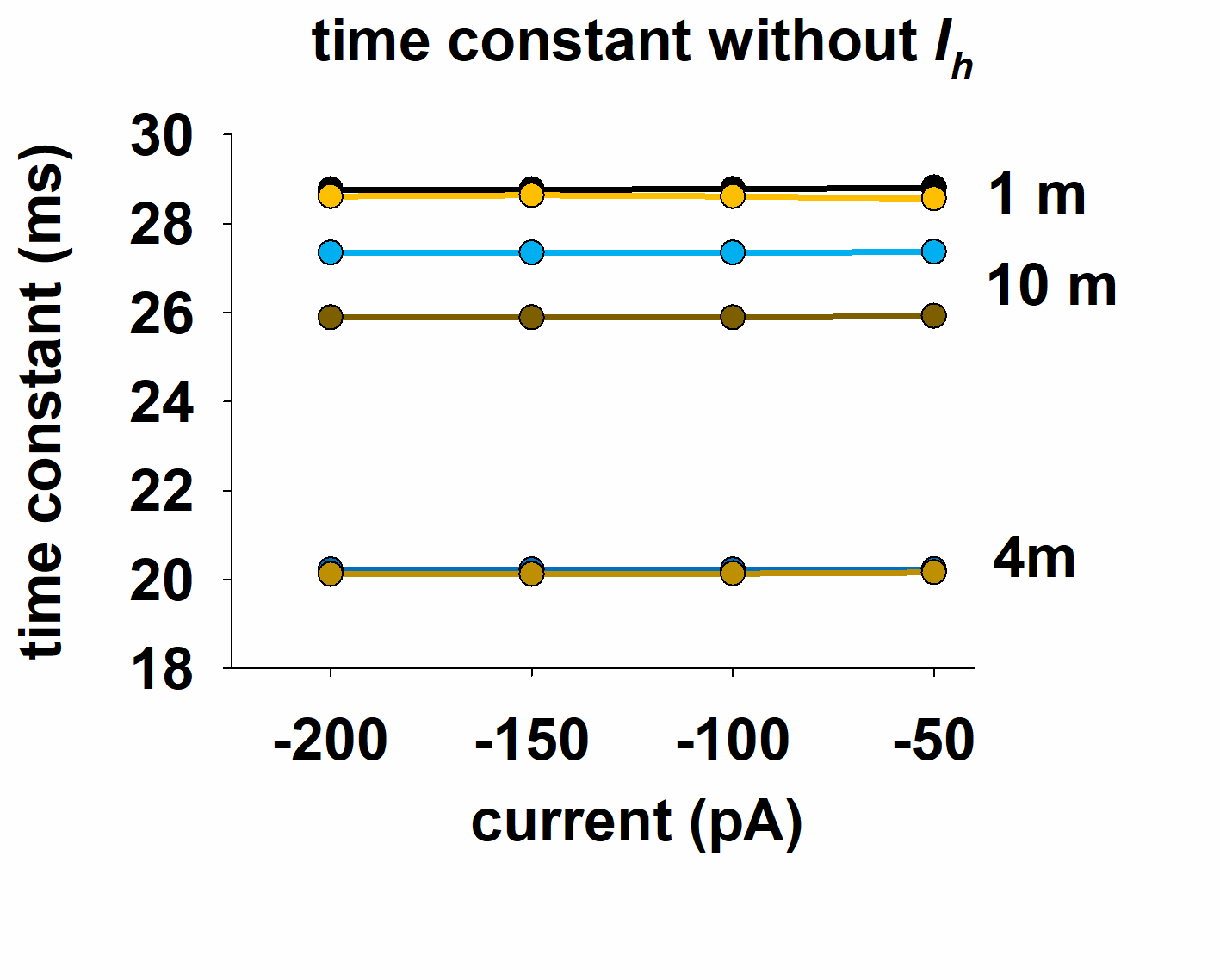


**Figure S1: A) Comparison between model traces for WT-10m mice with (*left*) and without (*right*) *I_h_* channel. B) Membrane time constant as a function of the current injection without *I_h_* (compare with Fig.2E).**
